# A Perspective on the Interplay of Ultraviolet-Radiation, Skin Microbiome and Skin Resident Memory TCRαβ+ Cells

**DOI:** 10.3389/fmed.2018.00166

**Published:** 2018-05-30

**Authors:** VijayKumar Patra, Léo Laoubi, Jean-François Nicolas, Marc Vocanson, Peter Wolf

**Affiliations:** ^1^Centre International de Recherche en Infectiologie, Institut National de la Santé et de la Recherche Médicale, U1111, Université Claude Bernard Lyon 1, Centre National de la Recherche Scientifique, UMR5308, Ecole Normale Supérieure de Lyon, Université de Lyon, Lyon, France; ^2^Center for Medical Research, Medical University of Graz, Graz, Austria; ^3^Research Unit for Photodermatology, Department of Dermatology, Medical University of Graz, Graz, Austria; ^4^Allergy and Clinical Immunology Department, Lyon Sud University Hospital, Pierre-Bénite, France

**Keywords:** skin microbiome, ultraviolet-radiation, skin resident memory T cells, inflammation, immune suppression, photomedicine, phototherapy

## Abstract

The human skin is known to be inhabited by diverse microbes, including bacteria, fungi, viruses, archaea, and mites. This microbiome exerts a protective role against infections by promoting immune development and inhibiting pathogenic microbes to colonize skin. One of the factors having an intense effect on the skin and its resident microbes is ultraviolet-radiation (UV-R). UV-R can promote or inhibit the growth of microbes on the skin and modulate the immune system which can be either favorable or harmful. Among potential UV-R targets, skin resident memory T cells (T_RM_) stand as well positioned immune cells at the forefront within the skin. Both CD4^+^ or CD8^+^ αβ T_RM_ cells residing permanently in peripheral tissues have been shown to play prominent roles in providing accelerated and long-lived specific immunity, tissue homeostasis, wound repair. Nevertheless, their response upon UV-R exposure or signals from microbiome are poorly understood compared to resident TCRγδ cells. Skin T_RM_ survive for long periods of time and are exposed to innumerable antigens during lifetime. The interplay of T_RM_ with skin residing microbes may be crucial in pathophysiology of various diseases including psoriasis, atopic dermatitis and polymorphic light eruption. In this article, we share our perspective about how UV-R may directly shape the persistence, phenotype, specificity, and function of skin T_RM_; and moreover, whether UV-R alters barrier function, leading to microbial-specific skin T_RM_, disrupting the healthy balance between skin microbiome and skin immune cells, and resulting in chronic inflammation and diseased skin.

## Introduction

### Skin microbiome

**Figure 1:**
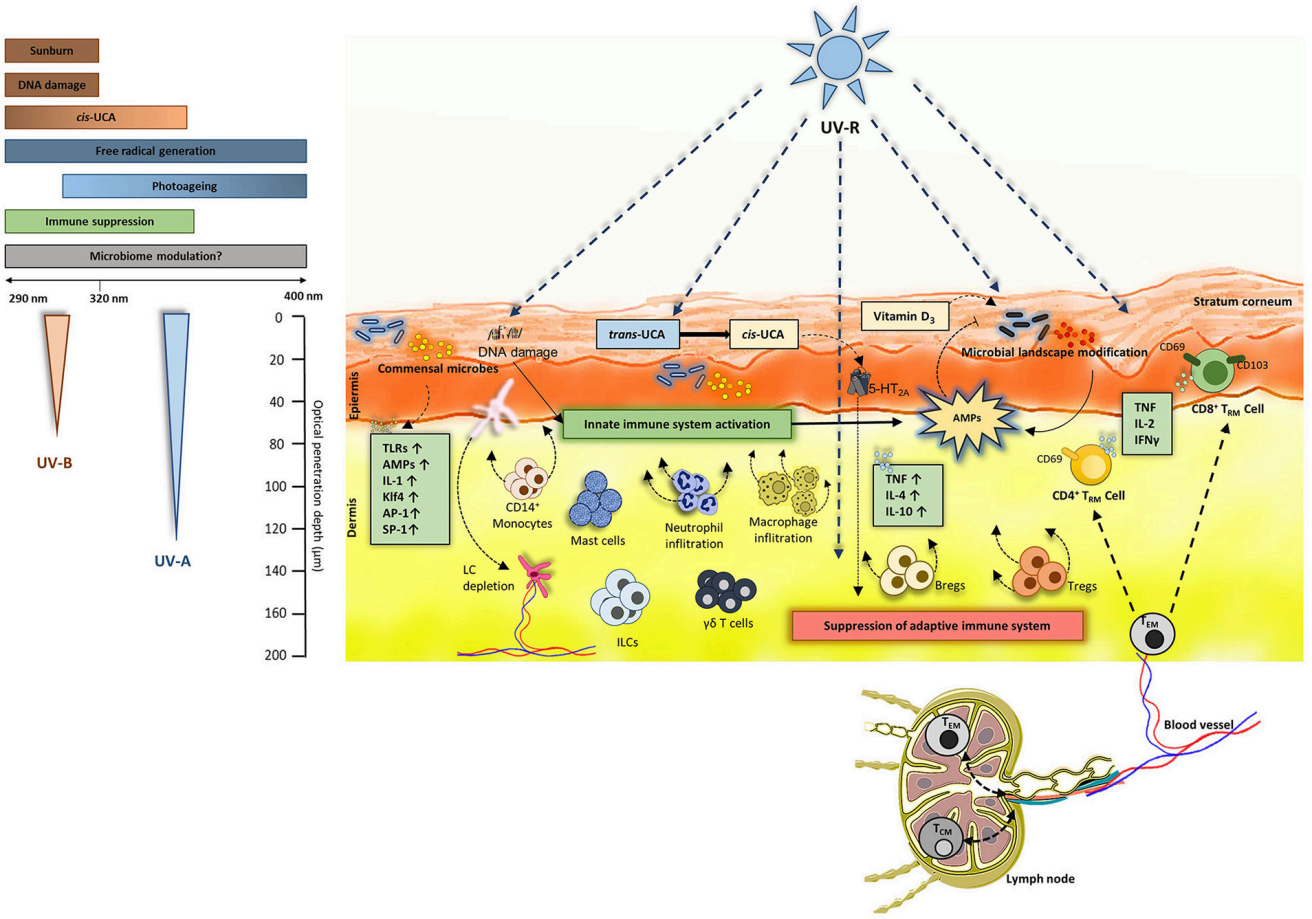
UV-induced events in the skin: Both UV-B (290–320 nm) and UV-A (320–400 nm) penetrate the skin. UV-B causes sunburn and DNA damage and is also known to induce immune suppression. UV-B and UV-A (to some extent) converts trans-UCA to cis-UCA and generates free radicals. Commensal microbiome colonizes the skin and can induce production of various cytokines, antimicrobial peptides (AMPs) and activate toll-like receptors (TLRs). The effects of UV-B and UV-A on skin microbiome is not fully understood. Overall, UV-R is known to activate innate immunity by production of AMPs and by stimulating innate cells like macrophages, mast cells, innate lymphoid cells (ILCs) and skin resident γδ T cells. On the other hand, UV-R induces an immune suppressive environment in the skin by inducing production of TNF, IL-4, IL-10. As overall result, regulatory T cells (Tregs) and B cells (Bregs) are induced leading to functional immune suppression and subsequent inhibition of effector T cells present in the skin. Regarding TCRαβ^+^ lymphocytes, effector memory T cells (T_EM_) can circulate between the blood, lymph and skin where they receive environmental signals. Also, the dermis is populated by CD4^+^ T_RM_ (CD69+ CD103±) whereas the epidermis is composed of CD8^+^ T_RM_ (CD69^+^CD103^±^) in majority. These T_RM_ populations can produce TNF-α, IL-2 and IFN-γ depending on the microenvironment. Nevertheless, the UV effect on these T_RM_ remains to uncovered.

Human skin with its large surface (1) harbors a wide variety of microbes, which include bacteria, fungi (2), viruses (3, 4), archaea (5, 6) and skin mites (4, 7, 8). These microbes exist in either a mutualistic and/or competitive relationship with each other (microbe-microbe) (9) and the host (10–13). Commensals make up for most of the microbiome followed by opportunistic and/or pathogenic microbes. The diverse physical nature of the skin with its variable water content, pH, lipids and sebum quantity among others crucially influence the diversity of the microbiome. However, it is intriguing that myriads of microbes reside on the skin surface (Figure 1) as well as in sub-epidermal compartments (14), despite the robust nature of the skin's immune system to rapidly detect and neutralize any foreign intruders (15). Many common cutaneous conditions such as atopic dermatitis (AD), psoriasis and rosacea are associated with dysbiosis of skin microbiome, most commonly driven by commensal species. A recent review highlights the latest findings regarding the microbial interactions with the immune system and microbial composition in health and diseases such as AD, acne, chronic wound infections, and primary immunodeficiencies (16).

### Ultraviolet-radiation (UV-R)

UV-R is one of the most prominent external factor affecting the skin (17) and the microbiome (8, 18, 19). UV-R mediated immune suppression was first discovered by Kripke et al. (20). This was further confirmed and proved to be T-cell mediated by using contact hypersensitivity (CHS) models in mice (21) and in humans (22–24). The initial key events that are prominently involved in immune suppression after UV-irradiation are DNA damage (25), formation of reactive biophospholipids like platelet activating factor (26) and isomerization of inactive *trans*- to active *cis*-urocanic acid (UCA) (27). A study conducted by Kubica et al. (28) used caspase-14 deficient mice which are known to have reduced levels of UCA and observed significant alterations in the skin microbiome. It is intriguing that caspase-14 is involved in proteolysis of filaggrin which is the major source of UCA in the skin and mutations in filaggrin are linked to the development of AD which is in turn linked to an altered microbial landscape (29). Certain skin commensals such as *Micrococcus luteus* can degrade cis-UCA to its trans isoform (30) and thus potentially diminish immune suppression. An early report from our group suggests that cis-UCA can indeed directly modulate skin microbiome (31). Since UV-R suppresses the immune reaction to antigens of infectious microbes such as *M. lepraemurium, bovis BCG, C. albicans, B. burgdorferi*, and *Schistosoma mansoni* (32–34) it can be speculated that exposure to UV-R could enhance susceptibility to infections, however clinical evidence of increased infections after UV-R is very low. This could be due to the fact that UV-R suppresses adaptive immunity but activates innate immunity (35). One of the important innate key players are antimicrobial peptides (AMPs). These are small proteins typically ranging from 10 to 50 amino acid residues that have potential to neutralize invading microorganisms (36) and mediate adaptive immune response (37–39). Dysregulation in AMP expression could be linked to many diseases, including photosensitive conditions like polymorphic light eruption (PLE) (40), where AMPs may be key mediators to maintain homeostasis between host immune system and microbiome. UV-R exposure also leads to infiltration of macrophages and neutrophils (41–43), induces emigration of Langerhans cells (LC) from the skin into the draining lymph nodes (44–46) and affects mast cells. Furthermore, regulatory T cells (Tregs) and B cells (Bregs) are recruited and activated (47, 48). All these cells and UV-induced events are known to be involved in immune suppression (49) (Figure 1). It has been known for a long time that UV-induced immune suppression is mediated by T cells (21, 50), however, the exact role of UV effects on the more recently described T_RM_ and immune function are largely unexplored.

### Skin-resident memory T cells (T_RM_)

**Figure 2:**
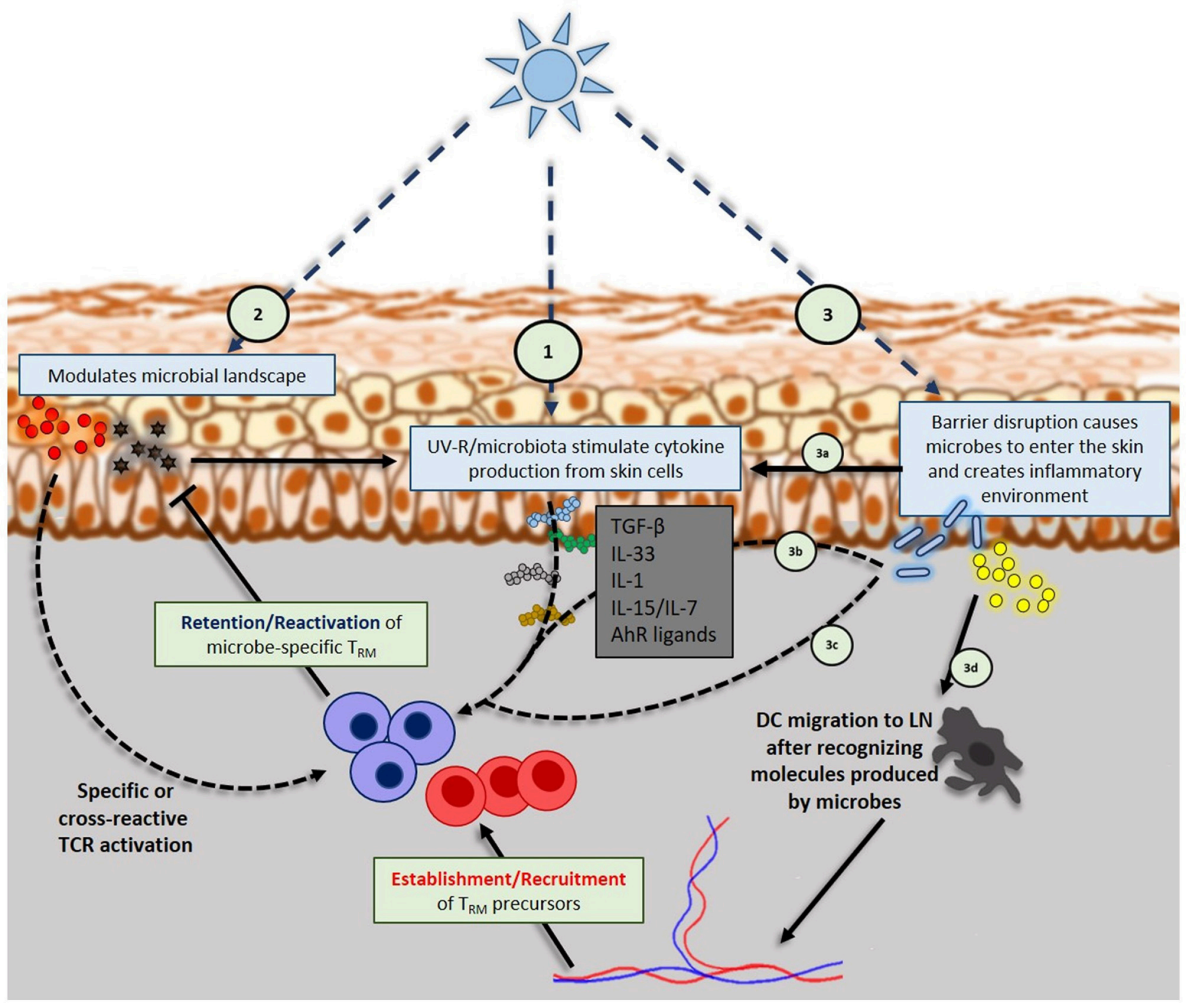
Interplay of UV-R, skin microbiome and skin resident memory TCRαβ+ cells: (1) UV-R induces keratinocytes and other skin cells to produce inflammatory or regulatory cytokines that will influence T_RM_ phenotype, retention and reactivation. (2) UV-R modulates microbial landscape, eventually releasing microbial antigens into the skin that will be up taken by dendritic cells (DC) that will specifically activate T_RM_ (regulatory or effector). Microbial antigens can also trigger the production of inflammatory cytokines by keratinocytes that further activate T_RM_. (3) High doses of UV-R can cause barrier disruption that will allow skin resident microbes to enter the skin; danger signals from barrier disruption (3a) and microbes entered into the skin (3b) will trigger cytokines production by keratinocytes, DCs, ILCs, NK and TCRγδ cells. Those cytokines will take part in shaping T_RM_ phenotype and activation. Entered microbes can also activate skin T_RM_ in a specific manner (3c) or be uptaken by DCs (3d) in order to activate naïve specific T cells in draining lymph nodes that will be recruited on the site.

Among all the immune cells present in the skin, such as dendritic cells, macrophages, γδ T cells and NK cells, T_RM_ (51) are now considered as key players of immunity (52–54) (Figure 1). They have been described in various tissues such as skin, lung, gut, liver and brain (55–57). T_RM_, along with effector and central memory T cells (58), are either CD4^+^ or CD8^+^ T cells that are derived from naïve specific T cells which were activated upon a previous immune response. Thus, T_RM_ share a common clonal origin with central memory T cells (59) but diverge in terms of dynamics, phenotype, and function. The major characteristics of T_RM_ are their capacity to survive and stay poised in the skin for a long time (60) as well as play a key role for pathogen clearance and immune alert (53). In other words, T_RM_ do not recirculate in the lymph or blood but rather patrol in the skin. CD8^+^ T_RM_ are more localized in epidermis whereas CD4^+^ T_RM_ populate preferentially the dermis (61). This non-recirculating pattern is conferred by the expression of CD69 which blocks sphingosine-1-phosphate receptor (S1P1), a receptor normally allowing lymph entrance. Moreover, a significant part of skin T_RM_ express CD103, the α-chain of the integrin αEβ7 which interacts with E-cadherin expressed by keratinocytes. Once arrived in the skin, killer-cell lectin like receptor G1 (KLRG1)-T_RM_ precursors receive key signals for their establishment in the tissue. Among them, TGF-β is a critical signal integrated by T_RM_ via TGF-βRII (52) and required for their residency. TGF-β can notably be produced by keratinocytes which thus play a role on T_RM_ retention (62). TGF-β alone is not sufficient for skin T_RM_ establishment, but rather acts in combination with other cytokines expressed in the skin such as TNF-α and interleukin (IL)-33 (63). Moreover, hair follicles seem to play a role on the recruitment and establishment of skin T_RM_ notably through the production of IL-15 and IL-7 (Figure 2) (64). Apart from cytokines, lipids available in the skin are key for T_RM_ maintenance (65). Functionally, T_RM_ allow a faster immune response upon pathogen entry through the production of alarmins such as IFN-γ and chemokines to recruit neutrophils, monocytes as well as circulating memory T cells on the site. T_RM_ are also able to proliferate locally after a recall response to maintain themselves (66). Finally, T_RM_ are able to be strongly cytotoxic (67).

## UV-induced impact on skin TRM

At least 1–2 × 10^10^ resident T cells comprising T_RM_ populate the human skin (68, 69), and it is highly logical that they experience similar impacts from UV-R as the other immune cells. These sentinel cells have numerous essential functions within the skin for cutaneous immunity and repair along with wound healing, antimicrobial responses and local tissue inspection (68, 70–72). The impact of UV-R on immune response mediated by T cells such as CD4^+^, CD8^+^, and Tregs has been previously described (73–75), however, the effects of UV-R on shaping the persistence, phenotype and specificity of skin T_RM_ are poorly understood. It is therefore important to understand the interaction between the skin T_RM_ and UV-R in mediating UV-induced immune suppression. It is thought that after an acute UV-exposure, the damaged keratinocytes release ATP (76) and ATP-mediated IL-1 (77) in an accelerated way; furthermore, this extracellular ATP is thought to be involved in adaptive immune responses (78, 79). Moreover, UV-R upregulates CD69 expression on TCRγδ cells (77) and could exert a similar effect on skin T_RM_ for which CD69 is crucial for their residency in the tissue. Besides, in the absence of γδ T cells, there was reduced DNA repair of UV-induced lesions in mice, suggesting the role of these γδ T cells in the repair (77). Such a role for T_RM_ has been demonstrated in acute wounds (71) but needs to be addressed in the case of UV-induced damage. T_RM_ may have long been unknown targets of UV-phototherapy in diseases which are now understood as T_RM_ cell-mediated (80). Patients with cutaneous T cell lymphoma (mycosis fungoides) are known to have malignant T cells that lack L-selectin and CCR7 expression, a phenotype that is similar to T_RM_ (81). The common treatment modality for these patients include phototherapy (82) and low-dose radiation. However, the effects of phototherapy on T_RM_ is completely uncharacterized (83).

## Influence of skin microbiome on skin T_RM_

The skin is exposed to a large number of microbes throughout the lifetime, of which only a minor proportion is pathogenic. It has been suggested that the primary purpose of the immune cell memory is to maintain the immune homeostasis with the commensal microbes (84). Recent studies in various mouse models and in humans show that the composition of the skin microbiome is crucial in mediating appropriate immune responses toward a pathogen and in maintaining the normal immune status in the skin (10, 11, 15, 28, 85–87). Whether certain species of commensal microbiome influence the type of T_RM_ within the skin is not known, but a lot can be learnt from the gut. In one of the studies using mice, commensal specific memory T cells were found in the intestines (88) and similar T_RM_ cells could exist in the skin as well. Both memory CD4^+^ and CD8^+^ T cells can act against infections with influenza virus (55, 89), lymphocytic choriomeningitis virus (90, 91), herpes simplex virus (92), mycobacterium tuberculosis (93) and parasites (94). Furthermore, microbial and/or antigen-specific memory CD4^+^ and CD8^+^ T_RM_ cells produce vast amount of effector cytokines in response to microbes and antigens (95–97) and CD4^+^ and CD8^+^ T_RM_ cells can populate and persist in multiple tissue sites long after the microbe or the antigen has been neutralized (98, 99). In the skin, CD8^+^ T_RM_ can be generated following an infection (92, 100, 101) and CD4^+^ IL-17-producing T_RM_ cells were identified in the skin of the mice when they were infected by *C. albicans* (part of skin mycobiome) (102). Besides, another study showed that laboratory SPF (specific-pathogen free) mice had lower non-circulating T cells in the skin and other tissues compared to pet store mice (103). In terms of T-cell memory, SPF-raised mice have a similar adaptive immunity like newborn humans and pet store mice show the profile of memory T cells, similarly observed in adult humans (104). Several studies show a compartmentalization of microbe-specific memory T cells. When humans were injected intradermally with purified protein-derivative from *M. tuberculosis*, antigen-specific T cells were observed only in the skin but not in the blood (105). HSV2 specific CD8^+^ T cells were found in genital skin but not at other body sites (106). Variability within the skin microbiome (16) could be a reason for compartmentalization of T_RM_. Skin T_RM_ persists for long periods of time and are exposed to the microbiome and microbial antigens from the skin during their lifetime. Microbial-specific responses could be a part of the healthy immune balance between the skin microbiome and host immune system and further provide reinforced local immunity. Very interestingly a recent study demonstrated that non-invasive *S. epidermidis* allows specific CD8^+^ T_RM_ establishment through non-conventional MHC-Ib H2-M3 peptide presentation. Those H2-M3 restricted CD8^+^ T_RM_ were shown to play an important role in tissue repair and wound healing (107).

## Perspective

Skin microbiome and T_RM_ reside in the upper layers of the skin. Both UV-A and UV-B radiation can penetrate those upper layers (only UV-A particularly reaches the dermis) and imminently impact all the microbes and immune cells (Figure 1).

### Does UV-R directly shape the persistence, phenotype, specificity and function of skin T_RM_?

UV-R is known to induce production of various cytokines in the skin such as TNF-α (108) or IL-33 (109–111) which are known to be involved in maintaining the phenotype of T_RM_ (52, 64, 112). Besides, a study published in 2016 (62) linked UV-B exposure and T_RM_ retention. Authors demonstrated that UV-B exposure decreased αvβ6 and αvβ8 integrins expression by keratinocytes. Those integrins were required for active TGF-β production which then maintained CD103 expression on T_RM_ allowing their retention in the skin long time after a lymphocytic choriomeningitis viral infection. Hence, the ability of UV-R (notably UV-B) to dose-dependently influence the retention and phenotype of skin T_RM_ by modulating the cutaneous cytokine environment (Figure 2), certainly may at least contribute to the efficacy of suberythemal phototherapy, which has been used for decades to improve pathologies such as psoriasis, atopic dermatitis and other inflammatory diseases (113–116). However, beyond cytokines, it is also possible that T_RM_ persistence depends on TCR-specific signals. The discovery of commensal-specific T_RM_ in the gastrointestinal tract of mice (88) implies that there may be a large number of commensal-specific T_RM_ residing in the skin as well, in addition to γδ T cells, innate lymphoid cells and pathogen-specific T_RM_. Moreover, the skin microbiome is constantly changing within the individual throughout lifetime (117) and contributes to skin T_RM_ diversity and function (107). Interestingly, UV-R is known to influence the skin microbiome landscape (8, 18, 19, 31). UV may in a dose dependent fashion affect skin microbiome and may shape the repertoire diversity of effector or regulatory T_RM_. Important remaining questions are the contribution of T_RM_ to the local immune response against (i) non-specific, commensal microbes which could invade the skin upon a skin barrier damage and (ii) invading pathogenic microbes. The first question queries upon their role in chronic pathologies such as psoriasis, atopic dermatitis or PLE. The second question concerns the capacity of T_RM_ to provide a heterologous protection against diverse infections (118) (Figure 2).

### Does UV-R alter skin barrier function, further activating microbe-specific skin T_RM_ and causing chronic inflammation?

Commensal microbes are known to improve innate and adaptive responses by producing small molecules which act as mediators between the host and microbes (119). Recently it has been reported that commensal skin microbiome can modulate gene expression of various cytokines, TLRs and AMPs in total skin cells (120). In the skin *Staphylococcus aureus* is known to promote skin inflammation by producing phenol-soluble modulins (PSMs) (121) which can stimulate IL-1-type (IL-36α and IL-1 α) cytokine production (122) and IL-17 from dermal γδ T cells (123). Moreover, *S. aureus* secretes proteases which are involved in skin barrier damage, promoting bacterial penetration into the skin which could ultimately generate *S. aureus*-specific T_RM_ cells. A robust accumulation of commensal-specific T cells under defined conditions may lead to worsening pathogenic conditions such as psoriasis (124, 125). Psoriasis and AD are intriguing examples of possible T_RM_ interplay with commensal microbes. An inflammatory environment exists in these chronic diseases which may lead to severe barrier disruptions through the patient's life. This could eventually lead commensal microbes to penetrate the skin, produce microbial-antigens, and finally lead to specific T_RM_ recruitment and establishment at the inflammatory site. In this context, both allergen-specific T_RM_ and commensal microbe-specific T_RM_ are in place. Whether commensal-specific T_RM_ cells portray a regulatory role or participate in the inflammatory loop is not known. Commensal-specific T_RM_ may also play a role in PLE, an inflammatory skin condition in which itchy skin lesions of diverse morphology occur when the skin is exposed to sunlight. In this disease microbes residing on upper layers may be driven to induce the production of AMPs and express commensal associate molecular patterns (126) which could play a role in pathophysiology of the disease. Furthermore, the capacity of UV-R to cause a barrier defect (127) may contribute to this phenomenon. Patients developing PLE may have skin inhabiting or newly generated commensal-specific T_RM_ that get activated. An inflammatory microenvironment may lead to changes in microbial landscape, further increase specific T_RM_ activation and booster the inflammatory loop.

## Conclusion

The specificity of adaptive immune system is complexly linked to the establishment and the persistence of the T_RM_ which recognize previously encountered antigen via specific T cell receptors (TCRs). These specific T_RM_ are generated and kept as a pool of heterogenous population with respect to the numerous microbes and microbe-associated antigens that they encounter during the lifetime of individual. With recent discoveries about potential functions of skin microbiome to educate and modulate host-immune responses, it is important to identify how these microbes influence the skin T_RM_. Specifically targeting those T_RM_, directly or via microbiome may allow to develop novel treatment strategies, acting like or even better than phototherapy, but with an improved risk-safety profile.

## Author contributions

VP and LL: conceived the ideas and drafted the manuscript; VP: drafted the figures; J-FN, MV, and PW: corrected and contributed to the draft. All authors revised and approved the final version of the manuscript.

### Conflict of interest statement

The authors declare that the research was conducted in the absence of any commercial or financial relationships that could be construed as a potential conflict of interest.
